# Cultural and religious influences on Arab American healthcare experiences: a qualitative study

**DOI:** 10.3389/frhs.2026.1892061

**Published:** 2026-08-19

**Authors:** Mariam Aly, Julie C. Dunsmore

**Affiliations:** Department of Psychological, Health & Learning Sciences, University of Houston, Houston, TX, United States

**Keywords:** acculturation, Arab Americans, cultural responsiveness, culture, healthcare access, healthcare quality, immigrant health, religion

## Abstract

Immigrants negotiate aspects of their heritage and host cultures to adapt to their new social environment through the acculturation process. This process influences health and well-being in immigrant communities. Arab American immigrants in the United States face unique cultural dynamics that shape their experiences, including healthcare experiences. This study addresses a gap in knowledge about culture as a social determinant of health awareness, healthcare access, and healthcare quality among Arab Americans. Guided by acculturation theory, we conducted thematic analysis of semi-structured interviews with 20 Arab Americans. Themes emerged revealing that culture, religion, and family function as both facilitators and barriers to healthcare. Cultural norms often promote self-care and healthy behaviors, but may discourage seeking medical treatment, particularly for mental health concerns. Religion was described as a flexible influence on healthcare decisions. Participants preferred providers of similar ethnic backgrounds due to perceived cultural understanding, and participant recommendations for providers highlighted the need for improved cultural competency training among healthcare professionals. Research on culturally responsive healthcare and training and policy solutions that enhance providers’ cultural responsiveness are needed to improve healthcare experiences for Arab American patients and other immigrant populations.

## 1 Introduction

Healthcare providers in the United States (U.S.) play a vital role in improving health outcomes by delivering care that acknowledges and integrates patients' diverse ethnic and cultural backgrounds. Culturally responsive care has been shown to enhance communication, increase patient satisfaction, and promote more effective treatment adherence (1). This manuscript focuses on the healthcare experiences of Arab Americans in the U.S. There are 2.3 million Arab Americans living in the U.S (2)., yet barriers to healthcare access and quality from the unique perspectives of Arab American patients remain underexplored. As with other ethnic-cultural minority populations, acculturation may shape how Arab American patients navigate healthcare systems and impact both their health behaviors and healthcare experiences. Acculturation refers to the process through which individuals come into prolonged contact with an ethnically distinct dominant host culture. This process often involves immigrants and their descendants negotiating aspects of both of their heritage and host cultures to adapt to a new social environment. Through this negotiation, individuals strive to maintain a stable and consistent sense of self while balancing differing cultural expectations and values. These cultural adjustments, in turn, have a direct influence on health behaviors, perceptions of illness, and overall healthcare experiences (3).

We aim to explore negotiation of culture as a social determinant of health awareness and healthcare access and quality among Arab Americans in the U.S. using a qualitative approach that centers Arab American adults' perspectives of healthcare access and experiences. In doing so, we seek to contribute to the literature on developing interventions and policy solutions to improve healthcare for immigrants and their children. Findings of this study may have the potential to assist healthcare providers and policymakers to provide appropriate care for Arab American patients.

In the next section, we describe the background research and theory that informed the qualitative methodology. This background information includes research on culture and acculturative processes in relation to health outcomes in immigrant populations; an overview of socio-historical changes and common aspects of family and community life for Arab American in the U.S.; evidence of health disparities for Arab Americans in the U.S.; and aspects of Arab American social and cultural experiences that may be salient for the healthcare perspectives of Arab American adults.

## 2 Background research and theory

### 2.1 Culture and acculturation as social determinants of health among immigrants

The World Health Organization defines social determinants of health as “the conditions in which people are born, grow, work, live, and age, and the wider set of forces and systems shaping the conditions of daily life” (4), (p.1). Culturally-specific social norms and behaviors have sometimes been found to buffer against adverse health outcomes (5). For example, research on Mediterranean cultural factors has revealed that familial support and strong community bonds contributed to the resilience of Italian migrants in the U.S. town of Roseto, helping mitigate the negative health effects of socioeconomic deprivation (6). However, other studies discuss that cultural norms may also act as detrimental to health and well-being. In the instance of the Maltese population in Southern Europe, extended family relationships were sometimes perceived as controlling and harmful to participants’ mental and physical health despite being culturally valued (5). Similarly, Arab American culture tends to prioritize extended family over individual autonomy (7). Navigating cultural differences in the emphasis on family over individual autonomy will be part of the acculturation process that may affect the extent to which extended family influences play a protective role for health outcomes.

Acculturation may be conceptualized as an individual-level psychological process that involves how people adjust to living within a new cultural environment. Berry (8) proposed four acculturation strategies—assimilation, integration, separation, and marginalization—based on the extent to which individuals adopt elements of the host culture while maintaining their heritage culture. Within this framework, integration refers to a strategy in which individuals actively engage with the host culture while preserving key aspects of their original cultural identity. In contrast, marginalization occurs when individuals feel disconnected from both their heritage culture and the host culture. Integration generally corresponds with the most positive psychological adaptation, including higher self-esteem and better mental health, whereas marginalization is linked to emotional distress and identity confusion (8).

Research applying acculturation theory to Arab American communities reveals mixed findings. Some studies support acculturation theory in finding that greater host-culture immersion and interaction with beliefs is linked with improved psychosocial well-being and lower acculturative stress among Arab Americans (9). Other studies suggest that solely maintaining traditional Arab ethnic identity without integrating into the host culture relates to better psychosocial well-being and less acculturative stress (10).

It is important to note concerns regarding the portrayal of Arab ethnic identity. Firstly, the classification of Arab Americans as “White” within U.S. federal and public health data systems often leads to the aggregation of this population within broader racial categories. This practice limits the ability of researchers and policymakers to identify and analyze health disparities unique to Arab Americans. As a result, critical differences in disease prevalence, healthcare access, and social determinants of health remain obscured, contributing to gaps in health equity initiatives and targeted interventions (11). Secondly, many studies have portrayed Arab identity as a fixed or static cultural experience that conflicts with American cultural values. This perspective oversimplifies the complex nature of identity formation among Arab Americans, who navigate varying degrees of acculturation and generational differences. Moreover, much existing research has disregarded the diversity of national origins, languages, and sociopolitical contexts that shape lived experiences, overlooking the heterogeneity of the Arab American population. This risks reinforcing stereotypes rather than examining meaningful differences that influence health (12).

The qualitative approach of the current study respects the dynamic and heterogenous nature of Arab American experiences and prioritizes Arab American adults' own perspectives on salient aspects of their healthcare experiences. In summary, negotiation of cultures has important implications for healthcare experiences of minority and immigrant communities in Western multi-cultural societies, including Arab Americans.

### 2.2 Arab Americans in the U.S

Arab Americans face a unique combination of sociocultural circumstances that affect their cultural identities and integration into U.S. society. Arab Americans are a population with heritage from the Middle East and North Africa. Despite being legally classified as “White” in the U.S. due to historical sociopolitical contexts, many Arab Americans argue for recognition as a distinct ethnic group. This self-identification reflects their experiences as a minority group of color with unique cultural, social, and political realities. Research indicates that Arab Americans frequently report experiences of discrimination, stereotyping, and social marginalization, which are not captured by standard racial classifications (11, 12). These dynamics can have important implications for health outcomes, access to care, and the overall understanding of social determinants of health among this population.

Historically, Arab populations have been immigrating to the U.S. since the late 19th century through three major waves (9). The most recent phase began in the 1960s, due to more open immigration policies and an increase in educational and economic opportunities. Studies indicate that communities of Arab Americans from this more recent wave are more committed to preserving their ethnic and religious traditions and exhibit stronger nationalist beliefs, resulting in lower levels of assimilation compared to earlier generations (13). In recent years, the Arab American community in the U.S. has diversified in terms of national origin, reasons for immigrating, socioeconomic factors, and acculturation patterns. Members of this population now include individuals from countries like Yemen, Gulf countries, and North Africa (13). Many arrived for education, employment, family reunification, or as refugees from conflict. Furthermore, this diversity is reflected in variations in language proficiency, religious affiliation, levels of interaction with U.S. culture, and patterns of social integration, all of which shape their distinctive health needs and healthcare experiences.

Acculturative stress refers to the psychological strain that emerges due to challenges with adjusting to a new culture and has been recognized as a risk factor for both physical and mental health. While the Arab American community is highly heterogeneous, studies consistently highlight religion and family as primary protective factors of Arab ethnic identity against acculturative stress (9). In Arab home countries, direct instruction in religiously acceptable behavior is unnecessary because such norms are integrated into daily life (12). However, for both Christian and Muslim Arab Americans living in the U.S., religiosity serves as a baseline for immigrants to decide which aspects of American society to adopt or reject (14). Religion also functions as a source of resilience in withstanding acculturative pressures to engage in values or behaviors that contradict their ethnic traditions.

Family and community are also deeply interwoven with the Arab ethnic identity, reflecting the collectivist orientation of Arab cultures (15). Strong family and community networks have been associated with lower levels of depression and reduced acculturative stress among Arab Americans (9).

Higher socioeconomic status has been linked with greater adoption of American culture by improving English proficiency and increasing interaction with American society (9). Conversely, common risk factors can exacerbate stress for Arab Americans. Discrimination has been consistently documented as a key stressor, particularly after the attacks on September 11, 2001, which amplified anti-Arab sentiment and social marginalization (16). Notably, second generation Arab Americans have reported more frequent experiences of discrimination than first-generation immigrants, perhaps due to greater interactions with mainstream American society (10).

### 2.3 Health disparities faced by Arab Americans

Health disparities experienced by the Arab American population include greater risks of all-cause mortality, cardiovascular disease, diabetes, and cancer when compared to other White individuals in the U.S (17). Arab Americans face structural barriers such as higher uninsured rates, lower household incomes, and financial strain, all of which contribute to the underutilization of preventive health services and delayed healthcare-seeking behaviors (18). Furthermore, language barriers can impede effective communication with healthcare providers, reduce understanding of medical instructions, and limit access to preventive and specialty care (9). Cultural stigma and mistrust of the healthcare system further exacerbate these disparities, leading to delays in seeking care, lower adherence to medical recommendations, and increased psychological distress (19).

Regarding cultural stigma, a study examining providers' and Arab Americans patients' perceptions on diabetes treatment highlighted how cultural stigma associated with illness often discouraged Arab American patients from acknowledging their diagnosis or engaging in self-management behavior (20). Moreover, although nearly a quarter of Arab American adults report experiencing poor mental health, help-seeking is often restricted by cultural expectations tied to themes of honor and shame, especially concerning mental illness (21). As summarized by Ağılkaya Şahin (31), more religious individuals, including Muslims, may be less likely to seek professional mental health services. Individuals higher in religiosity may prefer clergy members or spiritual counselors over psychotherapists due to concerns that therapists may not respect their religiosity and religious practices, may not understand their beliefs, and may recommend solutions that conflict with their beliefs. These challenges reflect deeper cultural frameworks related to health, illness, recovery, and mortality.

### 2.4 Salient aspects of Arab American experiences for healthcare

Arab American patients construct holistic understandings of health informed by cultural beliefs and in relation to their social networks. Simultaneously, they actively influence their environment by shaping cultural practices within their families and communities. Moreover, healthcare systems themselves are embedded within broader political and societal forces and may reflect existing structural inequities. Understanding the lived experiences of Arab Americans in relation to navigating cultures and healthcare access and quality is important for culturally-sensitive healthcare. Literature suggests that differences between cultural and religious norms and contrasts between societal structures in the U.S. and those in Arab countries may be salient in Arab American perspectives about healthcare experiences.

#### 2.4.1 Differences between cultural and religious norms

One important social context Arab Americans perceive is being placed in the middle of two ways of life: the present-day Arab culture, and the Islamic way of life set forth in the Quran and Sunnah. While both ways of life share overlapping social norms and moral principles derived from social, political, and historical characteristics, they also diverge in key ways that influence identity, behavior, and social expectations (22). For instance, gender roles in many Arab societies are often informed by traditional and patriarchal cultural norms rather than Islamic doctrine, which in principle emphasizes spiritual equality between men and women. Consequently, certain social expectations within Arab communities, such as honor and shame dynamics and limitations on women's mobility, may be shaped more by cultural norms than by religious doctrine. This dynamic may create tension or negotiation between what is perceived as “cultural” vs. “religious” practice, and impacts how Arab Americans navigate daily life, family roles, and even health-related decisions within a U.S. context.

#### 2.4.2 Contrasts between societal structures in the U.S. and those in Arab Countries

Prior research shows Arab regions tend to differ from Western societies on cultural values such as collectivism (23), which holds important implications for healthcare perspectives, as cultural orientations toward collectivism can influence patient autonomy, family participation in medical decision-making, and expectations regarding provider–patient relationships. Furthermore, the acceptance of the Islamic religion has influenced educational and legal systems in the countries in the Arab world (24). This may shape healthcare systems through the integration of religious values into medical ethics, health policy, and the provision of care, including considerations such as gender interactions, end-of-life decision-making, and the permissibility of certain medical interventions (25).

One distinction of particular relevance to this paper is the difference of healthcare systems between the Arab region and the U.S. In contrast to the U.S., Arab countries struggle with the decreasing role of government in financing the healthcare systems, leading to a shortage of available health services, primary care and specialist physicians, nurses, pharmacies, and administration staff, especially in small cities (26). Moreover, the patient-centered care performance of Middle Eastern countries has been considered poor compared to other western countries due to population growth and the increased demand on health services, lack of financial resources, administrative and organizational reasons, and instability from war (27). Additionally, whereas the U.S. has a multiplayer health insurance system, some Arab countries such as the Kingdom of Saudi Arabia operate using a national healthcare system and universal healthcare insurance for all citizens that provides comprehensive coverage of care, including prevention, ambulatory care and inpatient services (26). These structural and systemic contrasts may be reflected in participants' perspectives through their expectations of healthcare accessibility, government responsibility in service provision, and the role of equity and social obligation in the provision of healthcare services.

### 2.5 The current study

To elicit Arab American adults' perspectives on their healthcare experiences in the U.S., we conducted semi-structured health-related interviews with open-ended questions exploring participants' apprehension of health as a holistic concept, perceptions of healthcare access, and the factors that facilitated or hindered their engagement with healthcare services. We conducted qualitative analyses of interview responses to identify salient themes regarding Arab Americans' perspectives on access to and experiences with healthcare services.

## 3 Method

### 3.1 Participants

Participants were 20 Arab American adults (15 women, 5 men; M age = 41.8 years, SD = 10.7, range = 27 to 60 years) who completed an online survey and an in-depth structured interview with an Arab American woman experimenter (the first author). All participants had obtained at least one degree in higher education and 80% had obtained a Master's degree or higher. Participants reported the following countries of origin or ancestry: Egypt, Syria, Iraq, Tunisia, Lebanon, Jordan, Palestine, and the United Arab Emirates. Based on participants' age and length of residence in the U.S., 12 participants were first-generation Arab Americans and 8 participants were second-generation Arab Americans. The average length of residence in the U.S. for first-generation participants was 20.08 years (range = 9–30 years). Based on participants' interview responses, all participants identified as Muslim and/or engaged in Islamic religious practices.

Table 1 shows participants' responses to items addressing material deprivation and economic pressure (28). Together, these responses suggest that the sample experienced relatively low levels of financial strain. Table 2 shows descriptive statistics for participants' responses assessing Middle East North Africa (MENA) ethnic identity (29). The sample was relatively high in considering Arab American cultural affiliation to be important in their ethnic identity.

**Table 1:** Descriptive statistics for financial strain items.

Item Wording	Response Options (Percentage of Participants Selecting Option)
In the past 12 months, how often did you or your immediate family need but were unable to afford any of the following: medical care, specialty care, mental health care, medical devices or monitors, or prescription medication?	Never (70%) Rarely (15%) Sometimes (15%) Often (0%)
Please indicate the level of difficulty in paying bills for you or your immediate family.	1 = a great deal of difficulty (0%) 2 = some difficulty (5%) 3 = a small amount of difficulty (35%) 4 = no difficulty (60%)

**Table 2:** Descriptive statistics for ethnic identity.

Subscale	Mean	SD	Min	Max
MENA Cultural Affiliation	3.46	0.45	2.80	4
MENA Media Use	2.20	0.85	1.33	3.67
Multiculturalism	3.65	0.44	2.67	4

Participants rate each item on a 4-point scale, 1 = strongly disagree to 4 = strongly agree.

Participants were recruited through printed flyers distributed at local college campuses, mosques, and community centers. Eligibility criteria included: (i) self-identification as Arab American, (ii) age 25 years or older, (iii), residence in the U.S. for at least two years. Interested participants received a recruitment phone screening and completed an online eligibility survey through Qualtrics. All participants received a detailed explanation of the study's purpose, potential benefits, and procedures both verbally and in written form. Written informed consent was obtained prior to enrollment. After completing both the interview and the post-interview online survey, participants received a $20 Amazon e-gift card as compensation.

### 3.2 Procedures

This research was conducted in accordance with American Psychological Association standards for the ethical treatment of participants and was approved by our Institutional Review Board. Participants provided written and verbal consent for participation and audio recording. Semi-structured in-depth interviews were conducted either virtually via Zoom or in person at a public location. Prior to interview, participants provided written and verbal consent for participation and audio recording. A health-related interview questionnaire was developed using a culture-centered approach following an extensive literature review. Open-ended questions explored participants' apprehension of health as a holistic concept, perceptions of healthcare access, and the factors that facilitated or hindered their engagement with healthcare services. All interviews were audio-recorded, transcribed verbatim, and analyzed for emergent themes using a grounded theory approach. After completion of the interviews, demographic and ethnic identity information were collected via a short online survey.

### 3.3 Measures

#### 3.3.1 Demographic information

Participants reported demographics such as age, country of origin/ancestry, length of residence in the U.S., and education, and completed material deprivation and economic pressure items (28).

#### 3.3.2 Ethnic identity

Ethnic identity and cultural affiliation were assessed using the Middle East North Africa Identity Measure (MENA-IM; 29). On this questionnaire, participants rate their agreement with 11 items on a 4-point scale (1 = strongly disagree to 4 = strongly agree). There are three subscales: MENA cultural affiliation, MENA media use, and multiculturalism. A sample item for MENA cultural affiliation is “Many things that are important to me are connected to my Arab American identity”.

### 3.4 Analysis

#### 3.4.1 Qualitative analysis process

Thematic analysis of the transcripts was conducted in four phases using a grounded-theory approach. A phenomenological lens was applied to emphasize participants' lived experiences of health and acculturation within the North American context. Analysis began with one researcher independently reviewing transcripts and assigning codes to segments of text. Initial codes were grouped into broader themes, and two additional researchers reviewed coding decisions throughout the process to assess inter-coder reliability. Disagreements were resolved through discussion until consensus was reached. During the second and third phases, subthemes were refined and confirmed. Final theme and subtheme names were selected through this iterative coding process in which codes and categories were continually refined to ensure that theme labels represented participants' communication about their lived experiences and, when possible, reflected participants' own language.

#### 3.4.2 Researcher positionality

The research team for the current study includes the two authors and two undergraduate research assistants who assisted with transcribing and coding interviews. The first author, who is bilingual in Arabic and English and identifies as Arab American and Muslim, led the data collection and interviewing process as well as the thematic analysis process. This shared linguistic and cultural background allowed participants to feel comfortable sharing their lived experiences and expressing emotions and concepts in Arabic. The second author, an expert in cultural influences on developmental and family processes, supervised the study's overall design, recruitment and analysis process. The second author identifies as White American and did not participate in qualitative coding beyond offering general guidance and supervision to the first author. The first research assistant was bilingual in Arabic and English and identified as Arab American and Muslim. The second research assistant identified as Muslim American and was bilingual in Urdu and English. Both research assistants contributed to qualitative coding, including interpreting findings and understanding the study's broader implications.

Coders' ethno-religious and linguistic backgrounds provided valuable cultural insights but may have also influenced their interpretations of the data. To minimize cultural bias and enhance reflexivity, the first author and research assistants engaged in bracketing practices throughout the study. They documented preconceptions and potential assumptions about the study population before and during data collection and analysis to ensure that interpretations remained grounded in participants' narratives rather than researchers' prior beliefs or expectations. Final study themes were agreed upon by consensus of the first author and the two research assistants during the coding process.

## 4 Results

### 4.1 Overview of domains

Study findings were organized into three main domains that emerged from the thematic analysis: general health, healthcare access and availability, and healthcare quality. The domain of general health reflects participants' multidimensional understanding of health and well-being, shaped by personal, cultural, religious, and familial influences. Participants frequently noted that health is not limited to physical functioning but represents a broad, holistic construct including mental health and health consciousness. As P9 explained, health encompasses “not just the physical health, but also the social, mental, and the spiritual health”.

Healthcare access and availability discusses the structural, cultural, and personal factors that facilitated or hindered participant's use of healthcare services. Finally, healthcare quality describes the conditions that shaped participant's satisfaction with the care they received including provider communication, cultural sensitivity and perceived competence. The sections that follow provide an in-depth examination of each domain and its corresponding subthemes.

### 4.2 Domain I: general health

#### 4.2.1 Culture as a promoter of general health

Findings revealed that culture plays a multifaceted role in the health of Arab American patients, promoting certain health behaviors while discouraging others. Participants described cultural norms that encourage healthy habits, emphasize self-care and social connection, and support maintaining an active daily lifestyle. Many framed these values as part of a broader cultural orientation toward collective well-being and a shared responsibility for family health. As P4 reflected, “First, I take care of my health for the sake of Allah (God), and then, second, is my culture… how I was raised, as health is a priority. So things are not left untreated.” Further sample quotes illustrating subthemes within Domain I may be found in Table 3.

**Table 3:** Subthemes and example quotes for domain I: general health.

Subtheme	Example Quotes
Culture as a promoter of general health	
General	Arab American can mean many things… but when it comes to healthcare, I feel as though we're kind of set up for success. A lot of our parents want us to be doctors, so we think, “Oh, doctors are healthcare, so we have to be healthy.” (P8)
Healthy dietary habits	The Mediterranean diet is considered a very healthy diet. The whole olive oil, the kebabs..everything is homemade and more on the organic side. The older generations are very big on eating fruit, especially for my kids. (P9)
Encouragement of self-care and physical activity	As Arabs, sports are embedded in our culture… Everyone in Egypt from a very young age practices a sport. (P11) Taking time for meditation, taking time for rest… there is background in the culture that emphasizes those things. And the same thing with social health. (P15)
Use of natural remedies for treatment	My two-month-old has acid reflux, and my dad he's a doctor said, “I can prescribe you some medicine, or you can do what my grandma did: make cumin tea.” If it's a cold, I'll make tea with honey, lemon, and black seed. (P9)
Culture as a limiter of general health	
Discouragement of healthy habits	Health is slowly being understood by the younger generation, but the older generation… you'll get comments like, ‘Why are you exercising if you're thin?’ If I don't drink sugar in my tea to avoid extra sugars, they say, “Enjoy it while you can. You're young and you're thin.” (P10) Health awareness is better on the higher end of the standard of living… but people with lower standards of living don't go to the doctor, they don't know, they don't care… because there are other factors they believe are more important, like making money or feeding a family. (P13)
Unhealthy leisure and dietary habits	All of my dad's friends are dying of heart issues… although they go to the smoke all night. (P18) Traditional food in Egypt is quite healthy… but you can always make healthy food unhealthy with pounds of butter and ghee. Egg rolls, samosas… when you look at the impact of eating such delicious food on a daily basis. (P14)
Stigma related to mental health	We try to overlook the anxieties and depression that our community is facing … even though it's more common in our community. But back when I was younger, mental health was something like, “Oh, they were probably possessed by a jinn (Arabic word for demon).” (P9)
Religion as a promoter of general health	
Taking care of one's health as a form of worship	My religion taught me you have to take care of your health because Allah one day is going to ask you, “What did you do about your health?” Our body is an amana (entrustment from God). You can't just neglect yourself. (P1) Our body is a gift from God … we have to treat it very well and appreciate it as something precious. If you are practicing the religion the right way, taking care of your health should be a priority. (P5) I was raised as a good Muslim… I have to take care of my health and my body to be part of the community, to volunteer, to do my job correctly. You're more impactful as a healthy Muslim vs. a sick Muslim who has to rely on others. (P6)
Religious encouragement of healthy dietary habits	It's prescribed that whenever you eat, you don't eat until you're full. That's narrated very well from Prophet Muhammad. Even with drinking water, you don't drink it all at once; you drink it in bits to allow your stomach to take what you're giving it. (P16) There are stories of the Prophet where he rarely ate red meat. There's actually a hadith supporting that you should probably not eat a lot of red meat. (P9) I try to add honey to my diet, and honey is something mentioned in the Qur'an. Anytime I'm not feeling too well, I add a few black seeds into my tea and that makes me feel good. That is from the Sunnah, from the beloved Prophet. (P11)
Religious fasting	Muslims, for example, we fast Ramadan, and Ramadan is by itself a definition of how to be healthy. It's actually a prescription for a healthy lifestyle… Western culture now defines intermittent fasting, and many studies show how it helps with controlling diabetes, hypertension, and cholesterol. The rituals of Islam actually established this stuff long ago, more than 1,400 years ago. (P13)
Encouragement of physical activity	Praying five times a day is almost like yoga positions that they've discovered. (P18)
Religion as a way to cope with stress	When I'm stressed out, I usually increase my prayer and remembrance of God … The positive affirmation in our religion brings a lot of peace inside you … it makes you feel that somebody is caring for you and watching over you. (P12)
Religion discouraging unhealthy habits	I don't drink. I don't smoke. This is part of how I respect my religion, because it guides you to be better and healthier. So as a Muslim, I feel our religion is helping us and protecting us to be healthier. (P11)
Family as a promoter of general health	
Encouragement of healthy habits	My kids always encourage me, “You have to have your own time, Mom. Take a break and go with your friends.”(P6) We have a treadmill at home and encourage one another to go on it … even family activities, we try for it to be something where we get some physical activity. Even my dad calls and tells me about the different vitamins I need to take. (P14)
Encouragement of seeking medical treatment	I grew up in a family where we do at least the annual checkup … My daughters panicked when I told them I stopped my cholesterol medication and said, “No, please take your medication. Do it for us.” (P16)
Taking care of one's health for the sake of their family	As a new father … the first imitation our child will look up to is us. If we're not physically active or if we're eating junk food and telling them “don't do that,” they won't listen. (P9) You have to take care of yourself because if I don't take care of myself, how am I going to take care of them? So knowing that I personally am the caretaker of the family makes me feel like I have a responsibility to take care of my health so that…I can take care of my family. (P4)
Family as a limiter of general health	
Caretaking responsibilities	I'm very busy with my family. And in our culture, the mom or the lady in the house does most things… so my family may hold me back. I don't have enough time to exercise as I plan to. I don't have time to go to the doctor and take care of myself on top of everything. (P6)
Encouragement of unhealthy habits	Because we're such a generous community … when I was trying to cut down on what I'm eating, to my mom that was a sign of disrespect … … that I only got half a plate of food or didn't get seconds. And so that can also hinder my health.” (P11)
Encouragement of not seeking medical attention	My family is way more likely to promote not taking medication or say, “Hey, I've got some extra pills, do you need them?” … Even with things like checking for breast cancer, they look at it like, “Oh, it hasn't been adopted. Why would I do something like this? Is this really a problem over here?” (P19) “Don't go to the doctor. I've got some antibiotic from overseas” … and it's usually some expired thing they picked up on the street. (P5)

Participants frequently emphasized the high regard for physicians within Arab culture. Several noted that medicine is a respected profession and that this respect reinforces positive attitudes toward health and healthcare. Another prominent topic involved the role of the Mediterranean diet and traditional food practices. Participants described “back home” diets as healthier and more natural, with limited exposure to processed foods and more organic food in the Middle East. Participants also emphasized the cultural importance of physical activity and movement. Many discussed sports as embedded in childhood routines or described daily walking as part of everyday life overseas.

Self-care emerged as another cultural value, expressed through grooming, appearance, and practices that promote emotional regulation, such as mental rest and reflection as cultural practices. Finally, many participants discussed the cultural use of natural remedies as a first line of care. These practices were described as part of cultural identity and as a preferred approach before seeking formal medical treatment. Participants acknowledged that in many Middle Eastern countries, preventive healthcare is less emphasized, and patients instead rely on self-care and home remedies. Interestingly, this reliance on self-care and home remedies existed alongside a deep cultural respect for physicians and medicine, highlighting a seemingly paradoxical relation in which professional medical expertise is highly valued, yet formal healthcare may be delayed in favor of managing illness within the home first.

Collectively, these narratives show how Arab culture promotes general health through dietary norms, physical activity, respect for medical expertise, self-care, and natural healing practices. These culturally rooted behaviors contribute to participants' holistic understanding of health and shape their everyday health habits in the U.S. context.

#### 4.2.2 Culture as a limiter of general health

While many cultural norms were described as supportive of health, participants also identified cultural practices and generational patterns that hinder health or discourage engagement in healthy behaviors. A common theme involved differences in health awareness across generations, with many participants noting that older Arabs tend to be less health-conscious than younger generations.

Several participants felt that this lack of awareness was even more pronounced among Arabs living in the Middle East. P11 described, “The health culture back home, from a generic perspective, is very low… people's health awareness is not very strong in Egypt. People care more about the social life than taking care of their own health.”

Participants also highlighted socioeconomic status as an important factor shaping cultural health practices and knowledge. Another cultural practice described as detrimental to health was the prevalence of smoking and hookah, especially among older Arabs. This normalization of tobacco use within social gatherings was viewed as culturally embedded yet harmful.

Unhealthy dietary habits also emerged as a prominent theme. While participants acknowledged that the Mediterranean diet is inherently healthy, many explained that cultural cooking methods can make traditional foods unhealthy. Furthermore, cultural expectations around hospitality and overeating during social gatherings were also seen as contributing to weight gain and related health concerns.

Finally, participants discussed cultural tendencies to overlook or minimize mental health concerns. Such interpretations were described as barriers to acknowledging and treating mental health conditions that may be communicated or accentuated through family relationships. Collectively, these narratives highlight how cultural norms surrounding food, social practices, and generational attitudes can limit general health and impede engagement in preventive or health-promoting behaviors.

#### 4.2.3 Religion as a promoter of general health

Religious values also played a central role, in shaping participant's understanding of general health. Many participants described Islam as a faith that encourages self-care, moderation, and proactive attention to well-being. As P9 noted, “If people follow the religion, the religion is always promoting health in all aspects, physical and emotional. Being good with relationships.” See Table 3 for additional sample quotes.

Participants frequently emphasized that caring for one's body is viewed as a religious duty, rooted in the belief that health is a sacred trust from God. Others echoed this sense of stewardship by describing the body as a divine gift.

Another prominent theme involved the belief that maintaining good health allows individuals to be more productive and beneficial to their communities. Similarly, P7 referenced a Prophetic saying, “the strong believer is better than the weak believer”, to emphasize that strength and vitality enable one to contribute more effectively to others.

One common subtheme involved dietary practices informed by Islamic guidance, which participants viewed as promoting moderation, balance, and physical well-being. Several participants referenced well-known prophetic sayings about limiting food intake. P8 highlighted this principle, sharing, “Our Prophet (PBUH) stated, ‘One-third of the stomach should be for food, one-third for water, and one-third for air’… so don't get too full on any of those things. That's a big deal.” Participants also described prophetic examples related to nutritional choices, such as limiting red meat and reinforcing dietary moderation, while others noted incorporating foods mentioned in religious texts into their health routines. Furthermore, participants also highlighted the health benefits of fasting, particularly during Ramadan, as another religious practice that promotes well-being. For many participants, fasting was perceived not only as an act of worship but also as a practice aligned with contemporary health research.

Another prominent subtheme involved the physical benefits embedded in daily prayer. Participants viewed the structured, repetitive motions of prayer as promoting flexibility, circulation, and regular physical movement throughout the day.

Beyond physical health, participants emphasized the role of religion in fostering emotional resilience and mental well-being. Several described Islam as encouraging optimism, positivity, and emotional regulation. Religious coping emerged as a prominent strategy participants used to manage stress. Others further expressed that religion provides emotional security and a sense of protection, helping them endure stress and restore equilibrium.

Participants also identified religious prohibitions, particularly avoidance of smoking and alcohol consumption, as important promoters of general health. These prohibitions were viewed not only as spiritual obligations but also as practical measures that protect physical well-being.

These narratives illustrate how participants viewed Islam as a powerful promoter of physical, mental, and spiritual well-being. It is important to note that participants also acknowledged that some health-limiting behaviors arise not from religious teachings themselves but from “cultural Islam”, patterns and interpretations that diverge from the true health-oriented essence of the faith.

#### 4.2.4 Family as a promoter of general health

Family influences were woven throughout participants' narratives, with family members modeling health behaviors, reinforcing expectations, and shaping everyday perceptions of wellness. Participants emphasized the strength of familial ties in Arab culture and noted that shared health beliefs often emerge within households. As P4 described, “We share the same vision about health… We're mindful of our nutrition, value staying active, and enforce habits that are good for our well-being.”

Families played a central role in encouraging the adoption of healthy routines, including self-care and mental health practices. Participants also discussed the role of family members promoting preventive care and adherence to medical treatment.

Another prominent theme revealed was participants feeling a responsibility to maintain their health for the sake of their families. These reflections demonstrate that family acts as a powerful promoter of general health, shaping attitudes, behaviors, and motivations across generations within Arab American households.

#### 4.2.5 Family as a limiter of general health

While family played a largely supportive role for most participants, a subset (25%) described ways in which family dynamics could hinder their ability to maintain good health. These limitations were often linked to time constraints, cultural expectations, and norms surrounding care, hospitality, and medical decision-making. Sample quotes are in Table 3.

Several participants explained that family obligations, particularly caregiving responsibilities, left limited time for self-care. Others described how cultural hospitality within Arab families could unintentionally undermine healthy eating habits. Participants also noted instances where family members discouraged appropriate medical care or reluctance towards medical guidance, perhaps reflecting the influence of community norms on family members' responses.

Importantly, many participants clarified that while family responsibilities can create practical barriers, these limitations are often unintentional and tied to life circumstances rather than a lack of support. Many emphasized that with effective time management, family obligations and personal health can coexist. As P5 stated, “If a person knows how to manage their time properly, then taking care of the family and staying healthy should not contradict.”

Together, these narratives highlight the complex ways in which family life, while deeply valued in the Arab culture, can sometimes create constraints on health behaviors, especially within the context of cultural expectations and caregiving roles.

### 4.3 Domain II: healthcare accessibility and availability

#### 4.3.1 Provider's similar cultural background as a promoter

Participants frequently expressed a preference for healthcare providers who shared their cultural, ethnic, or religious background, describing such relationships as fostering greater comfort and mutual understanding. Several participants described feeling more at ease with Arab or Muslim providers due to shared language and familiarity with healthcare practices in their countries of origin. P4 explained her relationship with her Arab primary care physician (PCP): “It's just the feeling or belief that she'd give me the top of the gear care. Maybe because we share the same language and beliefs, I feel she's more aware of my treatment history because she was part of the healthcare system in my country. So, an Arab or Muslim doctor is closer to understanding me and what I'm looking for in my treatment.” Further sample quotes illustrating subthemes within Domain II are in Table 4.

**Table 4:** Subthemes and example quotes for domain II: healthcare accessibility.

Subtheme	Example Quotes
Provider's similar cultural background	With my current primary care physician, I do feel a bit of an added level of collegiality with her because we both come from North Africa and I think our relationships to like the men in our lives are similar and our expectations as women and in our lives and our families are similar and stuff. And it was just kind of like a feeling, like a vibe in the air. I felt safe with her and I also respected her like expertise because she was like she is top of her field. And so I personally took pride in that and I felt really comfortable having her give me care (P16). Sometimes doctors who are not my religion, but they are from a different culture … like India, Pakistan, Hispanic cultures … … I feel we share a common culture, values, and family. (P10) I worked with this one Muslim Urologist back when I was in school … And so with urology, many male patients are not open about their Urological health to begin with … And almost every time I was in clinic, I had one at least one patient tell me that if it had not been for this doctor and the way he can kind of connect with them, they would not have pursued this certain treatment. So I think it makes a massive difference for a lot of patients. (P20) There are some health problems that are more common in people from the Middle East. When the provider is from the same background, he knows what's common in our population so it makes me feel like I'm in good hands… A lot of health problems come from dietary issues so having a doctor from the same culture would help so much, you know, to understand why I'm coming with that you know high cholesterol, for example, mainly because it's our diets that include certain types of food. (P12) If I were to go to a counselor or anything psychological, it matters a lot because a non-Arab or non-Muslim cannot relate. We have taboos. We have certain red lines that somebody who doesn't know your culture will immediately dismiss or don't relate at all. So I would say, for mental health, psychology, I need someone of my same culture. (P11)
Provider gender preference and access	Sometimes there's specific female providers who are known within the community for being accommodating with Muslim women. So, they're so popular that it's actually hard sometimes to get onto their caseload … And so there have been times where I've called an office or looked them up and it's only a male. And I'm like going down the list of what my insurance will cover, trying to find one that's female (P18)
Religion not perceived as a limiter to care	It's not religion that limits me from OBGYN exams … it's me being shy and hating anyone touching me. Religion teaches us that health is a necessity. Our religion is flexible. (P12) I went to a psychologist during a period of my life when I was anxious. I never felt like my religion told me not to seek help. (P15) In Islamic law, in medical cases it is allowed. If you are sick, you need treatment. I don't think because of my religion I am restricted. (P2)
Culture as a limiter to healthcare access	
Fear of disclosing symptoms	Arab patients fear that if they say their symptoms, they will have the same disease … But especially in the older generations, patients don't like to share all the details or they might confuse the doctor, “Where's the pain?” “Yeah, it's here, here, here, everywhere” … they have the tendency to say, “I'm not sick,’ when they are.” (P8) Arab patients lie a lot about their symptoms. They would go to the doctor sick, dying at home and they're like, “I'm doing fine.” … They get so excited about seeing the doctor and then when they get there everything goes down. (P5)
Fear of seeking medical treatment	Our perspective in Egypt is that only very sick people go to the doctor and if you go to the doctor, then that means that you're going to die. Similar with mental health. A lot of times in our culture, going to a mental health doctor used to be like, “oh, only crazy people go to the mental health doctor”. So now taking your son or your daughter to a mental health doctor because they have ADHD is very common here. They don't look down upon something that, but back in our culture, “No, no, don't take them to the doctor. Why take him to the doctor? He's fine.” (P11) I've met a lot of Hispanics who have this idea that they're going to be almost poisoned by the doctor. And again, the trust is much higher if it's a Hispanic doctor … My sister-in-law, she's Pakistani and she will only go to the doctor if she's like dying. And she'll just be like looking up remedies online and I'm like Google's not a doctor … but you know it's that that idea that we're not sure if we can trust those medical people and what they're selling us and what they're going to tell us … So minorities just distrust the whole thing. (P20)
Cultural masculinity norms as a limiter of healthcare access	There's a bit of a machismo culture… Arab men are expected to just keep going and have all the answers… and that could be a hindrance to seeking treatment… Especially with older people, it can turn into denial or putting something off. (P16)
Immigration as a limiter to healthcare access	When my husband came here… he had no idea what to access. At the beginning, when he wasn't a citizen and didn't have a green card, it was more just trying to access free clinics with barely any resources… they're able to give something, but if you need more specialized care, that's absolutely not accessible. (P18)

Participants also highlighted unspoken cultural understandings or implicit recognition as central to feeling safe and respected in clinical encounters. Importantly, participants noted that cultural concordance was not limited strictly to Arab or Muslim identity. Several described feeling similarly comfortable with providers from other minority or collectivist cultures that share comparable family values, social norms, and relational styles. These accounts suggest that perceived cultural similarity, rather than exact ethnic matching, can foster empathy, emotional attunement, and culturally responsive care.

Furthermore, participants described how culturally concordant providers reduced the need for self-advocacy and facilitated clearer communication, ultimately making healthcare easier to access and navigate. P18 described her experience with an Arab American Muslim primary care physician, stating: “There's a lot that just doesn't need to be said. There's a lot I don't have to advocate for because she assumes it … She already knows which way I'm going to be looking, and she's already done the footwork without me having to ask.” This implicit understanding reduced participants' burden to explain cultural preferences or contextual factors influencing their health decisions and promoted healthcare access. Participants also highlighted the importance of providers' familiarity with culturally specific health patterns and risk factors.

Several participants described how cultural knowledge was especially critical when religious practices intersected with medical care. P13 discussed fasting during Ramadan as an example: “If you have like a physician from the same culture, he or she would understand many examples of what you do on a daily basis that may contribute to your disease state. So for example, if you're trying to fast in Ramadan and you're diabetic … … if you have like a Non-Arab or non-Muslim provider, they may not understand the importance for you to fast like all day and they may not even know like because they haven't maybe seen this before or how to provide certain medication changes for the regimens throughout the day… ”.

Participants consistently reported that culturally responsive providers were better equipped to offer realistic, respectful treatment plans that aligned with patients' values rather than conflicting with them. This preference was particularly pronounced in the context of mental health care. Participants expressed concerns that providers unfamiliar with Arab or Muslim cultural frameworks might misunderstand family dynamics, religious values, or cultural taboos.

Overall, participants described culturally concordant or responsive providers as playing a critical role in promoting healthcare access by fostering trust, reducing communication barriers, anticipating cultural needs, and tailoring care in ways that respected both cultural and religious contexts.

#### 4.3.2 Provider gender preference and access considerations

While religion itself was not viewed as a barrier, participants described gender preferences for healthcare providers that were shaped by personal comfort, cultural norms, and religious values. These preferences occasionally complicated access to care, particularly when female providers were limited or in high demand. P1 described the logistical challenges of accommodating these preferences within the U.S. healthcare system: “I was looking for a Muslim woman dentist for my daughter… it's so hard sometimes because I have to research and call and double-check if this doctor is available. The same thing with my son because he prefers a male doctor.” Please see Table 4 for further sample quotes.

#### 4.3.3 Religion not perceived as a limiter to healthcare access

Participants unanimously rejected the notion that Islam restricted their access to healthcare, particularly in cases of illness or emergency, often citing Islamic jurisprudence that permits otherwise restricted interactions when health is at stake. Others emphasized the flexibility of religious practice in urgent contexts, noting a preference for gender-concordant care when possible but not at the expense of timely treatment: “I usually ask for a female doctor, especially for OBGYN things because of our religion… but I don’t think my health is affected negatively because of this. If you plan it ahead you can always have an appointment with a female doctor. However, when I was hospitalized, I didn't have the luxury to ask… in our religion, in times of emergency, there's always a waiver.”

Additionally, participants clarified that reluctance toward certain types of care, particularly reproductive or sexual health services, stemmed from personal discomfort rather than religious restriction. Notably, religion was also not viewed as a barrier to mental healthcare utilization.

Overall, participants described religion as a promoter rather than a barrier to healthcare access. While religiously influenced preferences, such as provider gender, Islam was consistently framed as flexible, health-affirming, and supportive of medical treatment across physical, mental, and emergency contexts.

#### 4.3.4 Culture as a limiter to healthcare access

Participants described several cultural norms and beliefs that limited healthcare access, particularly through stigma, symptom minimization, and delayed help-seeking behaviors. Many noted a cultural hesitancy toward engaging with the healthcare system unless symptoms became severe, which contributed to underutilization of preventive and early intervention services.

Participants explained that seeking care was often viewed as a last resort rather than a preventive measure. P19 reflected: “I think Arab patients are used to waiting to see how bad things are before they go. I think the more awareness you have, the more you reach out for preventative services.” Additional sample quotes are in Table 4.

Participants also discussed cultural norms around downplaying or minimizing symptoms, sometimes to the point of withholding information from healthcare providers. This reluctance to disclose symptoms was frequently linked to fear of being labeled as “sick,” particularly among older generations. Participants suggested that these behaviors may be rooted in limited exposure to preventive healthcare in their countries of origin. P18 reflected: “Arab patients come from a culture that don't have preventative healthcare. So many of them are just super anti-doctor or anti-medication.”

Mental health stigma emerged as a particularly salient barrier originating from wider community norms and imparted within family structures. Participants explained that in some cultural contexts, seeking mental healthcare was historically associated with severe illness or social shame. Participants emphasized that these beliefs contrast sharply with U.S. norms that normalize mental healthcare, especially for children and adolescents.

Finally, some participants contextualized these experiences within broader patterns of medical mistrust among minority communities. Collectively, these findings illustrate how cultural stigma, fear of diagnosis, limited preventive health norms, and mistrust of medical institutions interact to limit healthcare access among Arab American patients. These barriers operate alongside structural constraints, compounding delays in care and reducing engagement with preventive and mental health services.

#### 4.3.5 Cultural masculinity norms as a limiter of healthcare access

Participants identified cultural masculinity norms that limited healthcare access, particularly among Arab men. Participants explained that cultural expectations surrounding masculinity contributed to reluctance in acknowledging illness or vulnerability. P17 described how pride and social norms intersect to limit care-seeking behaviors: “The pride and the cultural norm for Arab males to kind of deal with things on their own or not complain about that stuff all of that kind of together … … I think it ultimately ends up in them coming off as not willing to talk about their problems or whatnot. And making it very difficult to kind of provide certain types of care”.

Participants emphasized how these expectations foster denial and delayed care, particularly among older men. Participants suggested that this cultural emphasis on strength and independence not only reduced engagement with preventive care but also delayed diagnosis and treatment, ultimately hindering timely access to healthcare services. These findings highlight how gendered cultural expectations intersect with health behaviors, reinforcing barriers to care within the broader sociocultural context.

#### 4.3.6 Immigration as a limiter to healthcare access

Participants described immigration-related structures as significant barriers to healthcare access, particularly during the early period following arrival in the U.S. Many reflected that unfamiliarity with the U.S. healthcare system, combined with rigid appointment-based structures, limited their ability to seek timely care. P4 described the initial difficulty of navigating a highly structured system: “In the beginning it was very hard… you have to follow the structure very routinely. Or at night you have to go to more expensive services, like the emergency room, for something that's not really an emergency… after many years here, I found other things that I can do to avoid unnecessary visits to more expensive places”.

This learning curve often resulted in delayed care or reliance on costly emergency services until participants gained familiarity with alternative options, such as urgent care or virtual visits. Other participants emphasized that immigration status itself further restricted access and limited eligibility narrowed healthcare options to under-resourced settings.

Participants highlighted that these structural barriers disproportionately affected newly arrived immigrants, often forcing them to rely on limited services that could not meet complex or chronic healthcare needs. These findings underscore how immigration status and system unfamiliarity intersect to restrict equitable access to healthcare services.

#### 4.3.7 Discrimination as a limiter to healthcare access

Participants described experiences of discrimination as a significant barrier to healthcare access, particularly among visibly Muslim individuals. Several Muslim women who wear the hijab reported feeling marginalized, dismissed, or treated unfairly within healthcare settings, which negatively influenced both the quality of care they received and their willingness to seek care in the future.

These experiences were often characterized by providers dismissing patients' concerns or questioning their credibility. P12 recounted a particularly distressing encounter in an emergency department, where she felt her medical knowledge and parental advocacy were disregarded:

“The doctor wanted to give my daughter the same antibiotic that didn't work for two days. She didn't pay attention to what I was saying, as if I were an ignorant individual.” P12 further described how the physician undermined her authority by speaking directly to her child and framing her advocacy as harmful: “When I left the room, she told my daughter that your mom wants you to have cancer because CAT scans cause cancer… Then the doctor made sure everybody knew that I was a troublemaker.” P12 reflected that she attributed the provider's behavior to religious bias: “I felt this was because I am a covered Muslim, and she did not respect my intellectual capabilities.”

For some participants, repeated or anticipated discrimination resulted in the avoidance of healthcare settings altogether. P18 explained how previous experiences shaped her family's healthcare-seeking behavior, particularly regarding geographic location: “Whenever we need doctors, we go to areas with high Muslim populations… We live close to an area that historically has been known as a KKK capital of [STATE NAME]. I'll rarely go with my children simply to avoid having a Hijabi walking in … Usually my husband will take our daughter and hope that the accent and Arab name won't cause trouble.” These accounts illustrate how perceived and experienced discrimination, particularly toward visibly Muslim women, acts as a powerful deterrent to healthcare access and influences long-term healthcare decision-making.

### 4.4 Domain III: healthcare quality

In addition to structural and cultural barriers to healthcare access, participants emphasized healthcare quality as a distinct and influential domain shaping their healthcare experiences. Quality of care was not solely defined by clinical outcomes, but also by interpersonal interactions, cultural sensitivity, perceived provider competence, and respect for patient concerns. Sample quotes are in Table 5.

**Table 5:** Subthemes and example quotes for domain III: healthcare quality.

Subtheme	Example Quotes
Cultural and religious competency of healthcare provider	You might find physicians who may not know these cultural differences, and they'll just treat everyone like a boilerplate or a cookie cutter. That's usually as an indicative of cultural insensitivity … I mean, if you don't know how to treat your patients or deal with them from their different backgrounds, then you're just prone to be like a failing physician over there because there is a big component that you actually ignored. (P10) Oh, you're fasting Ramadan? Oh you started Ramadan already?’ before I even mention it … When they have this understanding of culture, it really makes me happy. (P11) I feel that maybe my doctors have experienced a lot of Muslim patients, so they have an understanding… they'll close the door so if any man would pass by, you have that privacy. They understand what it means to be a covered woman… I've never had anyone question why I ask for a female provider. (P14)
Religious accommodation as a promoter of healthcare quality	I've found that the doctors I've been around respected my religion. They were like, “Oh ma'am, we're so sorry we don't have a longer gown.”… This is what I like about the U.S., people are more open and understanding now than before. (P1) If they see you wearing a headscarf, the cultural awareness is there and they don't offer a handshake. This is highly appreciated by Muslim women. Respecting patients’ boundaries is really good here. (P6)
Provider gender preference and access	You can't guarantee finding a female provider… When I was going to have my kids, I had to sign papers saying I would accept a male physician in an emergency. You understand that in emergencies there's no choice, but it's still difficult knowing there's no real alternative. (P18) For something like an endoscopy, asking for every person in the room to be female was tougher… On a small level, it's easy to ask. On others, it's not quite as simple. Basic things like knocking before entering the room if there is Muslim woman inside being missed makes you feel like they're not actually aware… My primary care doctor is good, but the broader system gets maybe a C. (P19)
Patient self-advocacy	Doctors should know some of the things that we're unable to do due to religious reasons. If I go to an OBGYN, I think they should be more culturally sensitive to what our practices are… They should know what kind of exam is appropriate. Especially when a young girl needs to see a doctor, these things matter. They need to find a way around it. (P3) Sometimes they don't respect your choices, which is sad because you really have to advocate. I remember my doctor saying, “No, this exam is something we have to do,” and I had to argue with them. If somebody does not advocate for themselves, this is horrible… because they don't realize this is her whole life. (P7) The therapist asked me if I date, and I said no, I'm Muslim. Then she asked if I ever want to hook up with someone, and I said no. She told me I needed to live a fuller life and that people should be having sex at least once a month. I was very offended. I always thought cultural competency was common sense, but I guess that's why they teach it. (P13)
Patient Recommendations	I think because Arabs are still considered white. But even under the Middle Eastern group itself there are subcategories including Iranians, Israelis, and North Africans… And so I think it'll be key for healthcare providers to ask more questions to learn more of the culture and really embrace it. Because as you know, the US history of treating minorities hasn't always been very delightful. (P9) I wish physicians from different cultures read more on the importance of gender differences and privacy for men and women and makes patients uncomfortable. There are many physicians who may not know the differences and they'll just treat everyone like a boilerplate or a cookie cutter. I'd like to see physicians be more culturally open. Taking online classes is not the solution. What helps is that if they get trained with someone else from that same culture and shadow them. (P13) Providers should ask patients more on what they're comfortable with, especially with Muslim women… They need to more aware that there is an added layer of privacy and respect for personal space and for the appearance, the showing of their bodies. (P15) I think learning … things like not extending your hand out to offer a handshake to a hijabi women because you may make them feel a little bit uncomfortable and flustered and you know they won't know what to do. (P17) I do wish that there was more cross-cultural training when it comes to mental health. There are cultural differences and collective trauma in how health is talked about. It's not that Arabs don't believe in depression it's that it's just discussed very, very, differently. And so I wish that there was actually more of that sort of training so that it would actually, I think, remove a lot of the stigma. from both sides. (P18)

#### 4.4.1 Cultural and religious competency of healthcare providers

Participants consistently highlighted the importance of cultural competency as a critical component of high-quality healthcare. Providers who demonstrated awareness of cultural norms, religious practices, gender preferences, and privacy concerns were perceived as more trustworthy and effective. Participants emphasized that culturally competent care went beyond clinical knowledge and required an understanding of how culture shapes comfort and health behaviors.

P13 emphasized the importance of recognizing gender norms and privacy expectations, stating: “I wish physicians from different cultures read more on the importance of gender differences and privacy for men and women and what applies and what makes patients uncomfortable. So they understand those cultural differences and they respect them.” Participants further explained that failing to acknowledge these differences often resulted in care that felt impersonal and culturally insensitive. Other participants discussed the value of providers understanding broader health disparities and religious practices.

Participants noted that cultural awareness was especially meaningful when demonstrated proactively by providers. Several participants acknowledged that comfort with providers also varied based on personal experiences, length of residence in the U.S., and familiarity with the healthcare system. Despite this, participants emphasized that cultural competence could be achieved regardless of a provider's background when there was openness, respect, and willingness to learn.

#### 4.4.2 Religious accommodation as a promoter of healthcare quality

Participants consistently described religious accommodation as an important contributor to their perception of high-quality healthcare, particularly modesty and gender boundaries. Several participants emphasized the importance of providers recognizing and actively accommodating religious boundaries. P9 described advocating for these needs with her primary care provider: “My PCP is Indian, which is very similar in culture with the Arab culture. So, for example, when it's time for actual physical touching, she knows my background, my religion. And so she goes, ‘Is it okay if I touch here?’ And she also brings another male nurse in the room as well… I had to inform her first and it was an uncomfortable conversation, but I felt like it was worth it.” Participants also noted that prior experience with Muslim patients enhanced providers' awareness and sensitivity.

Participants expressed strong appreciation for accommodations related to modesty and privacy. However, participants also described how the absence of religious accommodation, particularly limited access to same-gender providers, negatively affected their experiences and contributed to feelings of exclusion or mistrust.

Finally, participants emphasized that religious accommodation is especially critical for recently immigrated individuals or those with limited familiarity with the U.S. healthcare system. P17 reflected: “People who don't speak English or don't know the system might feel alienated. Something as simple as not offering a handshake or being mindful of a headscarf gives them comfort… They feel more seen and more willing to divulge information.”

Overall, participants emphasized that religious accommodation, when present, enhanced perceived quality of care by fostering dignity and respect. Conversely, its absence reinforced feelings of marginalization, particularly in complex medical settings.

#### 4.4.3 Patient self-advocacy

Participants emphasized the importance of patient self-advocacy as a critical factor in ensuring healthcare quality. Many described the need to actively assert their preferences and boundaries, particularly within a healthcare system they perceived as highly standardized and, at times, culturally insensitive. For example, P2 highlighted the necessity of being proactive in protecting one's rights within healthcare encounters: “In healthcare you have to be proactive with your rights. Sometimes they push me to do things like birth control, and I keep saying no.” Participants noted that self-advocacy was especially important when healthcare recommendations conflicted with cultural or religious norms, particularly during intimate or reproductive health examinations. Others described how a lack of respect for patient autonomy often necessitated assertive self-advocacy.

Participants also described instances in which providers' assumptions or comments caused emotional harm, reinforcing the need for advocacy. P19 provided a detailed example of repeated pressure from healthcare providers regarding reproductive screening, despite clearly communicated religious and personal decisions: “As a Muslim woman, I'm not sexually active, and I feel like I constantly get pushed, ‘you still don't want a pap smear?’ I had to explain that I've spoken to female Muslim physicians, I've had the HPV vaccine, and I understand my risk. Only after asserting myself did they finally respect it.”

Overall, participants framed patient advocacy as both empowering and burdensome. While self-advocacy allowed them to protect their religious, cultural, and personal values, many felt it should not be the patient's sole responsibility to educate providers or defend their decisions. Instead, participants emphasized the need for greater cultural competence, respect for autonomy, and provider awareness to reduce the necessity for constant self-advocacy.

#### 4.4.4 Patient recommendations

Lastly, participants offered several recommendations to improve healthcare experiences and quality of care, with a strong emphasis on the need for greater cultural awareness and responsiveness within the healthcare system. Across interviews, participants consistently highlighted cross-cultural training as a critical strategy to address differences in how health is understood and treated among Arab patients.

Many participants emphasized the importance of increasing provider knowledge of cultural and generational barriers that contribute to stigma surrounding healthcare utilization. At the same time, they pointed out that stigma may also exist on the provider side, shaping assumptions about Arab patients and influencing care delivery. As P16 explained, “I think there might be a misunderstanding that Arabs might be antagonistic towards like healthcare authorities or following recommendations … But we Arabs and Muslims would like to clear up that generally we're taught to respect healthcare authority and take care of our bodies.”

Furthermore, participants stressed the need for providers to better understand the cultural and historical significance of healthcare within Arab communities. P19 highlighted how this awareness could transform provider–patient communication: “I wish they knew how much healthcare can mean to us. Because if they appreciate how much we value healthcare and have contributed to research on healthcare in the past … I think that would really change how they even communicated with people.”

Another key recommendation cautioned against treating Arab or Middle Eastern patients as a homogenous group. Instead, participants encouraged providers to recognize the diversity within these populations and to engage in more individualized, culturally informed care.

Additionally, participants suggested concrete ways by which their providers can engage more actively with patients by asking questions about their preferences, addressing language barriers, and being mindful of specific cultural norms around physical interactions. Training in cross-cultural communication and use of professional medical interpreters to limit language barriers may be scalable solutions that would address participants' recommendations. Overall, participants' recommendations highlight the need for a more culturally responsive healthcare system that moves beyond superficial cultural competency training and prioritizes understanding, communication, and respect.

## 5 Discussion

This study examined perspectives on healthcare experiences among Arab Americans, revealing a complex interplay of cultural, religious, and systemic factors that shape both healthcare access and healthcare quality for Arab Americans in the U.S. Findings indicate that culture and family play a multifaceted role in influencing health behaviors, simultaneously promoting some positive practices while discouraging others. Notably, religion emerged primarily as a facilitator of general health, encouraging individuals to seek care and prioritize well-being. The health-facilitating roles of religion and family are consistent with prior research (9), as is the recognition of stigmatizing cultural attitudes about health care and particularly mental health care (21). A key contribution of this study is the identification of culture's dual role as both a barrier and a promoter of healthcare access: while cultural stigma, masculinity norms, and distrust may discourage care-seeking, religious beliefs and family support can promote engagement with healthcare services. Furthermore, although family relationships were sometimes mentioned as limiters of health, this may reflect the role of families as mediators of societal expectations. In other words, broader cultural and community norms that limit health through stigma, delayed help-seeking, and healthcare decision-making may be transmitted or reinforced through family structures, while simultaneously families may function as important sources of support and encouragement for healthcare engagement.

Healthcare access was further influenced by immigration-related challenges, including difficulties navigating the healthcare system and limited awareness of available services. Furthermore, consistent with past research documenting discrimination experienced by Arab Americans (11, 12), participants described the detrimental impact of experiences of discrimination within healthcare settings. Perceptions of healthcare quality were shaped by provider cultural competence, shared cultural background or gender, and provider religious accommodation, underscoring the importance of culturally responsive care and the frequent need for patient self-advocacy. Findings suggest the importance of both systemic cultural responsive training and individual providers' skills and attitudes in the healthcare experiences of Arab American patients.

These findings can be understood through the lens of acculturation, highlighting how Arab immigrants uniquely navigate healthcare systems compared to broader immigrant populations. Prior research presents mixed perspectives on acculturation and ethnic identity among Arab Americans. Some studies suggest that greater immersion in the host culture is associated with improved psychosocial well-being and lower acculturative stress (9), while others find that maintaining a strong connection to Arab identity serves a protective role (30). Our findings suggest that Arab American healthcare experiences reflect elements of several of Berry's acculturation strategies rather than a single pathway. Participants who described greater comfort navigating the healthcare system, communicating with providers, and utilizing preventive services often demonstrated characteristics of integration, adapting to U.S. healthcare structures while maintaining important cultural and religious values. In contrast, some participants reflected aspects of separation, preferring providers from similar cultural backgrounds, relying on healthcare practices from their countries of origin, or avoiding certain services due to cultural norms and distrust of the U.S. healthcare system. Experiences of discrimination and feelings of alienation within healthcare settings also suggest that some individuals may experience elements of marginalization, in which barriers impede a sense of belonging within the healthcare system.

At the same time, participants' narratives demonstrated that acculturation is neither linear nor uniform. Distinctive aspects of Arab American experiences, including the strong role of religion in promoting healthcare engagement and the cultural emphasis on modesty, family, and collectivism, remained salient regardless of length of residence in the United States. Seemingly contradictory findings, such as the coexistence of deep cultural respect for medical professionals along with preference to manage medical issues through self-care and home remedies, may reflect these combined cultural values. Participants frequently described adapting to structural aspects of the healthcare system while retaining core cultural values, particularly regarding provider preferences, gender concordance, and religious accommodation. Furthermore, participants communicated that longer residence in the U.S., higher educational attainment, and greater exposure to healthcare systems were associated with fewer barriers to care and greater confidence navigating the healthcare system. Collectively, these findings support the notion that Arab American patients often engage in a dynamic process of selective integration, adopting aspects of the host healthcare system that facilitate access while preserving cultural and religious practices that remain central to their identity.

These findings have important implications for healthcare providers seeking to deliver more equitable and patient-centered care. Results highlight the need for culturally responsive care. Participants emphasized the value of experiential learning, suggesting that future providers train alongside diverse providers and patient populations rather than relying solely on online cultural awareness modules. Specific recommendations included asking patients about their preferences (e.g., provider gender, examinations, and communication style), avoiding assumptions about beliefs or behaviors, improving clarity when explaining diagnoses and treatment plans, and recognizing the importance of religion, modesty, and cultural values. Participants also underscored the need for greater access to culturally concordant providers to reduce the burden of patient self-advocacy, which was often necessary to receive appropriate care. Collectively, findings may inform potential interventions and policy strategies aimed at improving healthcare experiences for Arab American patients and other immigrant populations.

Strengths of this study include the focus on Arab Americans, an understudied population facing unique sociocultural circumstances in the U.S. Using a framework-based qualitative approach, the study captures the dynamic and heterogeneous nature of participants' healthcare experiences. The multifaceted themes that emerged are consistent with acculturation theory. Additionally, completing interviews with a researcher who shares an Arab American identity fostered trust and allowed participants to express themselves more openly, including expressing complex emotions and concepts in Arabic. Limitations include the small sample that was predominantly female and highly educated, which limits generalizability of findings. Furthermore, although the sample included both first- and second-generation Arab Americans, the small sample size limited examination of potential differences in acculturation and healthcare experiences according to generational status. Finally, all participants communicated Muslim religious affiliation and/or engagement in Islamic religious practices. Given the religious diversity within Arab American communities, it will be important for future research to address the ways in which Arab ethnic identity, religious identity, acculturation and individual experiences intersect to shape healthcare perceptions and behaviors.

Future research can build on these findings by longitudinally examining the role of acculturation in healthcare behaviors and experiences among Arab American populations over time. Additionally, expanding research to include more men, more diversity in religious affiliations and religiosity, and more diverse socioeconomic groups would improve generalizability and provide a more comprehensive understanding of within-group differences. Comparative studies with other immigrant and minority populations may also help contextualize healthcare experiences. Additionally, future studies should evaluate the effectiveness of cultural responsiveness interventions for healthcare professionals in improving patient–provider interactions and healthcare outcomes. Finally, exploring provider perspectives alongside patient experiences would further strengthen understanding of these dynamics and inform more culturally responsive care. Overall, findings contribute to the growing literature aimed at informing interventions and policy solutions to improve healthcare experiences for Arab American patients and other immigrant populations.

## Data Availability

The datasets presented in this article are not readily available because study participants did not give written consent for interview transcripts to be shared. Thus, research data is not available for sharing due to ethical considerations. Requests to access the datasets should be directed to Julie Dunsmore, jcdunsmo@cougarnet.uh.edu.
